# An Algorithm for Elective Amputation Combined with Targeted Muscle Reinnervation in Complex Regional Pain Syndrome—A Perspective

**DOI:** 10.3390/jpm12071169

**Published:** 2022-07-19

**Authors:** Martin Aman, Bahram Biglari, Mirjam Thielen, Arne H. Boecker, Annette Stolle, Daniel Schwarz, Emre Gazyakan, Ulrich Kneser, Leila Harhaus

**Affiliations:** 1 Department of Hand-, Plastic and Reconstructive Surgery, Burn Center, Department of Plastic and Hand Surgery, University of Heidelberg, BG Trauma Hospital Ludwigshafen, 67071 Ludwigshafen, Germany; martin.aman@bgu-ludwigshafen.de (M.A.); mirjam.thielen@bgu-ludwigshafen.de (M.T.); arnehendrik.boecker@bgu-ludwigshafen.de (A.H.B.); annette.stolle@bgu-ludwigshafen.de (A.S.); emre.gazyakan@bgu-ludwigshafen.de (E.G.); ulrich.kneser@bgu-ludwigshafen.de (U.K.); 2 Department of Paraplegia and Technical Orthopaedics, BG Trauma Center Ludwigshafen, 67071 Ludwigshafen, Germany; bahram.biglari@bgu-ludwigshafen.de; 3 Department of Neuroradiology, Neurological University Clinic, Heidelberg University Hospital, Im Neuenheimer Feld 400, 69120 Heidelberg, Germany; daniel.schwarz@med.uni-heidelberg.de

**Keywords:** CRPS, targeted muscle reinnervation, TMR, amputation, sudeck disease, nerve transfer, prosthesis, fMRI

## Abstract

Complex regional pain syndrome (CRPS) can result in a devastating condition. For a small number of patients, there is a non-response to any existing multimodal therapies and they ultimately request amputation. Such a drastic and final decision is not easy to take for both the patient and the surgeon and requires careful and interdisciplinary assessments and considerations. Furthermore, new surgical procedures, such as targeted muscle reinnervation (TMR) and hybrid prosthetic fitting, and multidisciplinary board advice should be included when considering amputation. In order to help other therapeutic teams in decision making for such rare but more than demanding cases, we aimed to propose an advanced algorithm for amputation indications in CRPS patients combining all these new factors. This algorithm consists of extensive pre-operative psychiatric assessment, diagnostic hybrid prosthetic fitting including fMRI analyses, multidisciplinary board advice as well as targeted muscle reinnervation and amputation procedures with final prosthetic fitting and rehabilitation. By involving multiple disciplines, this algorithm should provide optimized and individualized patient treatment on the one hand and a reliable base for decision making for therapists on the other.

## 1. Introduction

The final therapy-resistant stages of chronic complex regional pain syndrome (CRPS) are a devastating condition. Patients suffer from severe pain, trophic changes and loss of function, influencing all aspects of daily life as well as their psychological wellbeing [1]. Patients at this stage usually undergo a long lasting, partly frustrating multidisciplinary treatment approach involving not only physiotherapy and occupational therapy but also excessive pharmacological and psychological diagnostic and treatment. Even with advanced inpatient treatment, a small number of these patients do not recover from CRPS and become therapy resistant [2]. Some of these patients then request amputation of the affected extremity. As amputation is a severe surgical intervention with lifelong irreversible functional and psychological consequences and its effect on the course of CRPS is still under discussion, the indication of surgery must be considered carefully. Therefore, a multidisciplinary approach is indispensable, as patients are in an extreme and often desperate condition and may not be fully aware of potential consequences [3]. Further, recurrence of CRPS or the appearance of other chronic pain conditions after amputation (such as phantom limb pain, stump pain) may occur after amputation, which is a daunting condition for patients and surgeons. New surgical techniques such as targeted muscle reinnervation (TMR) are known to reduce phantom limb pain and neuroma formation after amputation [4]. Although gaining popularity, TMR is not commonly used in (elective) extremity amputation. 

We therefore aimed to propose an advanced algorithm, including these new surgical techniques and our experience with careful interdisciplinary preoperative board assessments to facilitate therapeutic decision making for elective amputation in cases of final-stage CRPS.

## 2. Background

### 2.1. CRPS and Amputation

Amputation is the most radical procedure which can be considered as a salvage strategy in therapy resistant CRPS. It may be suitable for patients who do not recover from CRPS after long term physiotherapy, occupational therapy, neuromodulation and extensive pharmacological pain treatment [2]. In fact, a recent study from Ayyaswamy et al. [5] showed that about 66% of the patients suffering from CRPS benefited from amputation with a general increase in quality of life. De Boer et al. [6] also found a significant improvement in the quality of life rating after amputation. Dielissen et al. [7] demonstrated an increase of 60% in function, although the extremity was amputated. Even though they could only demonstrate pain relief of 40%, the majority of the patients (85%) were satisfied with the outcome after surgery, thus showing that pain relief is not necessarily the main outcome after amputation. Patients also may see benefit in reduced anxiety and avoidance behavior due to the loss of the hyperpathic extremity [3,8].

Some authors hereby suggest that the main importance of amputation in CRPS is defining the level of amputation [7]. The level of amputation should be proximal to the level of allodynia to decrease the chance of recurrence. This is in contrast to the reports of Bodde et al., who proposed the level of amputation be secondary for the outcome and potential recurrence [8].

Ayyaswamy et al. [5] indicated that only 37% of the patients were using a prosthetic device after amputation due to recurrence of pain. Unfortunately, they were not able to differentiate between post-surgical pain, neuroma formation, recurrence of CRPS or phantom limb pain in terms of which aspect restrained people from using a prosthetic device.

### 2.2. Targeted Muscle Reinnervation

An innovative method to reduce phantom limb pain and neuroma formation is targeted muscle reinnervation (TMR). In this method, the transected nerves are transferred to residual muscles in the stump via selective nerve transfers. This has not only been proven to reduce occurrence of painful neuromas and phantom limb pain but furthermore to improve prosthetic function of bionic prostheses [9]. By reinnervating these muscles, more potential control signals can be created, resulting in superior prosthetic function and, therefore, higher patient satisfaction [10]. TMR can be performed on all levels of amputation in the upper and lower extremities. As amputations are performed mostly electively in such situations, and the affected extremities had only minor previous trauma, natural anatomy is preserved facilitating standard nerve transfer matrices for TMR. 

From a neurophysiological perspective, TMR leads to structural changes on all levels of the motor unit. By surgically transferring nerves to new target muscles, a hyperinnervation of the muscle or the distal target is created [11]. This is especially relevant for advanced prosthetic control, as the amputated limb should be replaced and the patient should be given the chance to have a functional improvement after amputation [12]. Structural changes in the central nervous system could also be observed [13]. Functional magnetic resonance imaging (fMRI) revealed an adapted cortical representation of the residual limbs after nerve transfers [14], which might also have a beneficial aspect in patients with CRPS.

The main fear in amputation planning is the conversion of CRPS into amputation-related pain syndromes. Some studies suggest that phantom limb pain occurs in up to 50% to 80% of patients after amputation [13,15,16,17]. Midbari et al. described a rate of 89% of phantom limb pain in their cohort of patients who underwent amputation after CRPS, which has to be examined in the context that the non-amputation controls reported higher pain scores and more impairment due to the pain and a lower quality of life compared to the patients which underwent amputation [18]. This aspect points out the high importance of phantom limb pain prophylaxis when performing amputation. Dumanian et al. demonstrated a significant improvement in phantom limb pain after TMR [19]. Especially in patients suffering from CRPS, care must be taken not to confuse phantom limb pain with recurrence of CRPS or postoperative pain of the stump. Therefore, we refer to the BUDAPEST criteria for detailed evaluation, but we are aware of potential overlaps and unclear cases [20].

### 2.3. Multidisciplinary Board

Multidisciplinary boards are nowadays common practice for complex situations such as those in oncology. Although gaining popularity, these boards are so far not common practice in other complex areas such as extremity surgery. In our unit, we established such a board for complex extremity reconstruction many years ago [21]. Furthermore, we run a specialized interdisciplinary CRPS outpatient clinic. By combining these two programs, we were able to set up an interdisciplinary CRPS board for complex cases and treatment questions. By involving all relevant medical disciplines, such as plastic and reconstructive surgery, orthopedic surgery, vascular surgery, rehabilitation medicine, pain therapists, physiotherapy, social services, prosthetists, psychiatry and psychology, the optimum treatment for the patient is discussed and decided together. This provides not only a profound basis for therapy but furthermore is important for medicolegal issues, especially for complex decisions such as elective amputation. Therefore, decision making should be carried out by all team members. 

### 2.4. Functional Magnetic Resonance Imaging (fMRI)

With fMRI, functional activation patterns of the affected extremity can be visualized for evaluation of the cortical representation of the limb.

Previous studies described alterations in fMRI in CRPS patients, such as a diminished representation of the affected extremity in the primary sensory and in the motor cortex. These alterations are not exclusively seen in CRPS but also in psychiatric disorders such as xenomelia and body integrity disorder [22,23,24]. Therefore, fMRI acts only as supportive data to visualize potential neglect and a long-time disuse of the affected extremity, which is usually accompanied by functional changes in the central nervous system. 

## 3. Advanced Algorithm for Amputation

As amputation is an irreversible lifelong consequence for the patient, precise patient selection is of utmost importance. In the decision making process, the first step should be to ensure non-response to any other therapy modalities for more than 2 years [25]. Therefore, it is most important to establish a complete report of pretreatment strategies and their outcome. Therapy options such as pharmaceutical as well as interdisciplinary pain treatments should have been exhausted. Although most patients have psychological treatment as part of their CRPS treatment, patients longing for amputation should be referred to a psychiatric assessment in advance of surgery planning. Patients should be assessed for psychiatric disorders, their ability to give informed consent as well as their awareness of the potential consequences of the amputation. This is also relevant for medicolegal issues. In case of any doubt, surgery should be postponed and psychological treatment should be assured [3,26]. Since CRPS patients have a higher risk for depressive, anxiety and post-traumatic stress disorder (PTSD) symptoms [1], existing psychiatric disorders may not automatically lead to a refusal of the amputation request but must be considered carefully in the decision making process. However, in conditions which impede informed decision making (such as acute psychosis or dementia) or significantly reduce the probability of successful aftercare, such as severe substance abuse, amputation should be denied. If any psychiatric disorder exists, it must be carefully weighed as to what impact the amputation will have on the psychiatric disorder and vice versa. In the course of this, coping strategies, psychosocial support at home and motivation for the amputation request should be assessed. The expected outcome should be discussed with the patient regarding his or her desired results, and any discrepancies must be addressed. 

In special cases of uncertainty, we refer our patients to the department of neuroradiology to assess the cortical representation of the affected limb in fMRI as an additional diagnostic tool. In these cases, the affected extremity is mostly neglected by the patient. With a follow up fMRI after the hybrid fitting of a prosthetic device, reactivation patterns in the area of the affected limb might be observed, indicating a further benefit from amputation for the patient.

Furthermore, to increase full awareness of potential consequences of amputation and prosthetic rehabilitation, our patients are referred to the prosthetist to create a hybrid fitting of the prosthesis. Thus, socket and prosthetic devices are created to be connected to the residual limb to give the patient an idea of future prosthetic replacement after amputation. As such, the patient is able to visualize the consequences of amputation and the potential functional outcome [26]. Hybrid fitting is indicated in any case, independent of additional fMRI.

**Figure 1:**
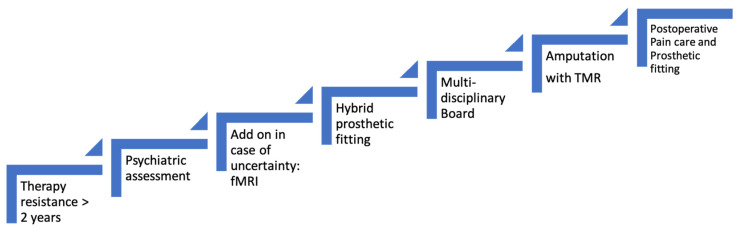
Algorithm for indicating amputation in CRPS patients.

When all assessments are completed and the results obtained, a multidisciplinary advisory board discussion regarding the amputation is then carried out (Figure 1).

Once these steps are taken, surgery can be planned. From our perspective, we prefer amputation at a level proximal to the level of allodynia. The incision should be planned to be extended and combined with TMR. An individual nerve transfer matrix is planned, depending on upper or lower extremity and level of amputation. We use the common nerve transfers shown in Table 1 [4,27]. Perioperative catheter placement is performed for regional anesthesia. Postoperatively, a consultant specializing in pain treatment is referred to the patient to ensure minimum post-operative pain and to minimize potential recurrence of CRPS. Additional psychological and supportive therapy, such as reduction of swelling, mirror therapy, stump desensitization etc. are also carried out.

After 6 weeks of wound healing and reduction of stump swelling, we then refer the patient to the prosthetist for prosthetic replacement. Hereafter, extensive prosthetic training in inpatient rehabilitation can be initiated to improve functional outcome and patient satisfaction with the new prosthesis. Furthermore, regular outpatient controls are necessary to ensure the best potential outcome and to be able to intervene directly if any signs of recurrence are present.

## 4. Algorithm Demonstration

**Figure 2:**
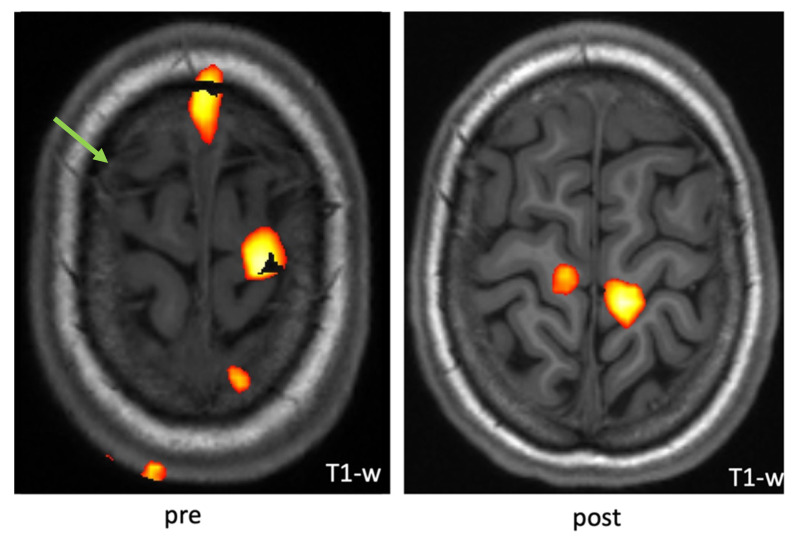
Example fMRI activation pattern upon a voluntary knee-bending task with the patient‘s right leg showing new co-activation of the right primary motor area. The green arrow indicates area of underrepresented activation while knee bending, whereas after hybrid fitting and training, new activation patterns could be observed.

For a better understanding of the proposed algorithm, we present its application on a 16-year-old, young female patient. Four years prior to amputation, she had a distortion trauma of the left knee and underwent arthroscopy. Just a few days after arthroscopy, she developed a fixed flexion contraction of the knee, which could not be improved with all possible therapies with and without anesthesia. Severe allodynia, increased hyperalgesia to cold, reduced body temperature of the affected extremity and increased hair growth as well as a neglect for the extremity followed as well as pressure sores and foot deformity due to constant sitting on the left foot. The progression of the foot deformity pointed towards a pronounced neglect of the affected extremity. Furthermore, the patient was diagnosed with anorexia nervosa, which developed during one of the inpatient pain treatments. After 4 years of extensive multimodal inpatient and outpatient treatment, which ended up as non-response to any therapy, the patient requested an amputation, supported by her parents. Their idea was to overcome the pain and the grotesque deformity of the leg, which was more than burdensome for her, resulting in her avoidance of any social contacts and school. After several outpatient consults in our clinic discussing amputation, we referred the patient to the psychiatrist, according to our algorithm. Psychiatry confirmed the patient’s and parents’ wish for amputation and her and her parents’ insight into the consequences. As the patient was of a very young age with a clinical neglect of her leg and had a history of the psychiatric disorder anorexia, we then referred her to the department of neuroradiology for fMRI analysis to obtain a further idea of the potential neglect of the extremity (Figure 2).

**Figure 3:**
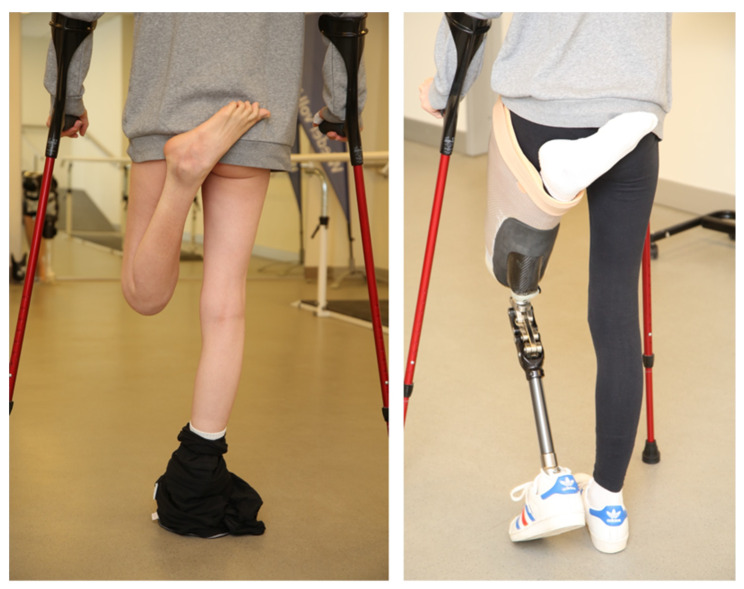
Algorithm demonstration on a 16-year-old female patient with CRPS of the left knee. She developed flexion contracture. After hybrid fitting of the prosthesis, she was able to experience walking with her prospective future prosthetic device.

We included this in our algorithm, as stated above, to further support indication for surgery with additional insights from cortical representation and activation patterns of the affected extremity. The fMRI indicated lower activation patterns of the affected extremity compared to the contralateral control. Afterwards, hybrid fitting of a prosthesis was initiated (Figure 3).

**Figure 4:**
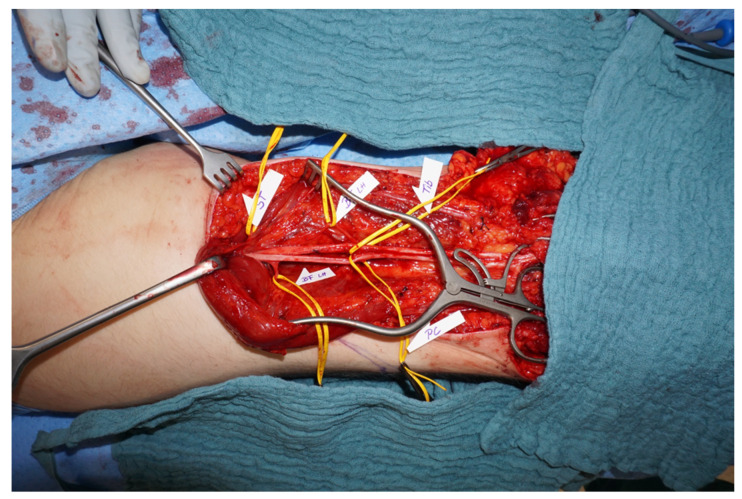
Amputation at above-knee level was performed in combination with TMR. Individual nerve transfers are displayed in main text and Table 1.

The patient used the prosthetic device for about 3 h a day over a 6-month period. We performed a second fMRI to evaluate cortical representation after prosthetic use, which showed a trend of increased cortical activation, further supporting the idea of a potential benefit of amputation and prosthetic replacement. A multidisciplinary board finally consented to amputation above the allodynia area of the knee. Operation was performed in combination with TMR of the sciatic nerve via transferring the tibial nerve to a motor branch of the semitendinosus muscle and the peroneal branch to a motor branch of the biceps femoris (Figure 4).

After wound stabilization, pain medication could be reduced significantly. The patient was fitted with a prosthetic device, which she is currently still using.

## 5. Challenges and Conclusions

We present an advanced algorithm which we use in our patients suffering from non-responding CRPS for elective amputation planning. As amputation is the last therapeutical option, a history of resistance to any other therapy options which are recommended for treatment of CRPS for at least two years and having a high level of suffering are requirements [25]. The expected outcome must be discussed with the patient with regard to his or her desired results and any discrepancies must be addressed.

Psychiatric evaluation ensures that the patients have full insight into the consequences of an amputation. This expert opinion is also important for medicolegal issues. FMRI can support an amputation decision by indicating reversible changes of limb neglect and limb disuse in the cortical representation. This tool thereby represents a non-compulsory examination, which might not be necessary in every patient. 

By fitting a hybrid prosthesis, patients cannot only see their future prosthetic device but furthermore test its function in daily life activities prior to surgery [26]. We strongly advocate hybrid fitting prior to surgery but are aware that, in certain cases, fitting a hybrid prosthesis prior to surgery might not be achievable due to intolerance of the patient.

Combining the amputation with TMR can not only decrease phantom limb pain and neuroma pain but also increase prosthetic function of a myoelectric prosthesis [9,19].

With a final prosthetic fitting and rehabilitation best potential outcome can be assured.

Further studies are necessary to prove the potential benefit of TMR in CRPS patients, although studies have described a high potential for decreasing phantom limb pain in patients suffering from traumatic amputations. Although current CRPS guidelines reflect that surgery in CRPS patients is theoretically possible (especially in type II), they do not state the value of amputation [29].

We think that this algorithm is helpful to decide for the complex indication of amputation in patients suffering from CRPS. The inclusion of new surgical techniques may help to improve the functional outcome and reduce recurrence and phantom limb pain after amputation.

## Figures and Tables

**Table 1:** TMR nerve transfer matrix according to different levels of amputation used in our facility. Note that individual planning is required according to definite level of amputation. The transfers were adapted from our experience from [4,27,28,29,30].

Level of Amputation.	Nerve	Targeted Muscle Motor Branch
**Glenohumeral Amputation**	Musculocutaneous	Clavicular part—pectoralis major
	Ulnar	Pectoralis minor
	Median	Sternocostal part—pectoralis major
	Radial	Abdominal part—pectoralis major Latissimus dorsi
	Deep radial branch	Infraspinatus
**Above Elbow Amputation**	Musculocutaneous	long head biceps brachii
	Ulnar	Short head biceps brachii
	Median	Brachialis
	Radial	Long head/medial head triceps brachii
	Deep branch of the radial nerve	Lateral head triceps brachii
	Deep branch of the radial nerve	Brachioradialis
**Below Elbow Amputation**	Median	Flexor digitorum superficialis
	Ulnar	Flexor carpi ulnaris
	Superficial branch of the radial nerve	Anterior interosseus nerve
**Above Knee Amputation**	Tibial	Semitendinosus
	Peroneal	Biceps femoris
	Posterior cutaneous nerve	Biceps femoris
	Saphenous	Vastus medialis
**Below Knee Amputation**	Posterior tibial nerve	Medial or lateral gastrocnemius
	Deep peroneal nerve	Tibialis anterior, peroneal mm.
	Superficial peroneal nerve	Peroneal mm.
	Saphenous nerve	Medial gastrocnemius
	Sural nerve	Tibialis posterior

## Data Availability

Not applicable.

## Abbreviations

CRPS	complex regional pain syndrome
fMRI	functional magnetic resonance imaging
PTSD	post-traumatic stress disorder
TMR	targeted muscle reinnervation
